# Deficits in Sustained Attention and Changes in Dopaminergic Protein Levels following Exposure to Proton Radiation Are Related to Basal Dopaminergic Function

**DOI:** 10.1371/journal.pone.0144556

**Published:** 2015-12-10

**Authors:** Catherine M. Davis, Kathleen L. DeCicco-Skinner, Robert D. Hienz

**Affiliations:** 1 Division of Behavioral Biology, Department of Psychiatry and Behavioral Sciences, The Johns Hopkins University School of Medicine, Baltimore, Maryland, United States of America; 2 Department of Biology, American University, Washington, District of Columbia, United States of America; Hudson Institute, AUSTRALIA

## Abstract

The current report assessed the effects of low-level proton irradiation in inbred adult male Fischer 344 and Lewis rats performing an analog of the human Psychomotor Vigilance Test (PVT), commonly utilized as an object risk assessment tool to quantify fatigue and sustained attention in laboratory, clinical, and operational settings. These strains were used to determine if genetic differences in dopaminergic function would impact radiation-induced deficits in sustained attention. Exposure to head-only proton irradiation (25 or 100 cGy) disrupted rPVT performance in a strain-specific manner, with 25 cGy-exposed Fischer 344 rats displaying the most severe deficits in sustained attention (i.e., decreased accuracy and increased premature responding); Lewis rats did not display behavioral deficits following radiation. Fischer 344 rats displayed greater tyrosine hydroxylase and dopamine transporter levels in the frontal cortex compared to the Lewis rats, even though radiation exposure increased both of these proteins in the Lewis rats only. Tyrosine hydroxylase was decreased in the parietal cortex of both rat strains following radiation exposure, regardless of proton dose. Strain-specific cytokine changes were also found in the frontal cortex, with the Lewis rats displaying increased levels of putative neurotrophic cytokines (e.g., CNTF). These data support the hypothesis that basal dopaminergic function impacts the severity of radiation-induced deficits in sustained attention.

## Introduction

Very little is known about the short- and long-term biological consequences associated with exposure to high energy and charge (HZE) and proton radiation. In addition to changing an astronaut’s risk of cancer, it is acknowledged that such radiation may have cumulative deleterious effects in multiple tissues, including the central nervous system (CNS). Ground-based studies demonstrate that radiation can induce behavioral changes in rodents, including impaired performance in motor tasks and deficits in spatial learning and memory [1–3]. Though these initial findings underscore potential dangers associated with radiation exposure, there is a limited understanding of the extent and degree of neurobehavioral alterations following exposure.

There is substantial evidence that suggests that dysfunction in the dopamine (DA) neurotransmitter system can contribute to impairment over a range of mission-critical CNS functions that include voluntary movement, feeding, reward, affect and motivation, sleep, working memory, learning, and attention [4–10]. Briefly, the DA system is highly sensitive to HZE radiation exposure, with measureable damage to DA neurons following both acute and chronic exposure (reviewed in [11–13]). HZE radiation can produce damage via direct particle strikes or focal lesions, via oxidative stress, and via microglial activation [11–13]. In this regard, DA cells are highly sensitive to both oxidative stress [14] and glial activation, including activation of astrocytes or microglia [14, 15]. Glial activation has also been demonstrated in the majority of diseases and disease models involving DA degeneration, including Parkinson’s disease [16–18] and Huntington’s disease [19]. HZE exposure can also damage DA systems in the substantia nigra and striatum, and produce deficits in DA-mediated behaviors [20–23]. Increased sensitivity to DA receptor antagonists following radiation exposure is also consistent with damage to the DA system [24]. Further, DA release from striatal slices is reduced following ^56^Fe exposure in rodents [20], but radiation from other ions/particles alters DA release as well [25, 26]. In sum, there is strong evidence of the adverse effects of radiation on the DA system, in addition to the behaviors regulated by DAergic activity.

Although many cognitive domains have been shown to be sensitive to DAergic disruption (e.g., impulsivity, reversal learning, spatial working memory), damage to the DA system produces well-characterized deficits in psychomotor speed, general motor function, and in fronto-striatally-mediated neuropsychological decision-making tasks [27–29], all of which are components in basic vigilance tests such as the human psychomotor vigilance test (PVT). Space analogue environments (including the Mars500 chamber simulation) and astronauts on board the International Space Station (ISS) currently use the human PVT (called the “reaction self-test” on the ISS) to assess performance readiness; tests similar to the human PVT are commonly used in the clinical setting to diagnose deficits in sustained attention that could result from fatigue, sleep-deprivation, or various psychiatric and neurological disorders [30, 31].

Employing a rodent version of the PVT, the rPVT, our laboratory has observed individual differences in the effects of proton radiation on neurobehavioral deficits and dopamine protein levels in outbred rats (i.e., rats that are not genetically altered or inbred; [32]). Since these individualized changes may be a function of radiation interacting with inherent biological differences, such as variations in basal DA tone prior to radiation, the current study assessed the effects of proton radiation on rPVT performances in inbred rats with differing basal DA tone and DA-related protein levels. The Lewis (LEW) rat strain displays a lower density of dopamine transporter (DAT) levels in the striatum, nucleus accumbens, and olfactory tubercle, compared to Fischer 344 (F344) rats [33, 34], as well as a lower density of DA D_2_ receptor levels in the striatum and nucleus accumbens of LEW rats [33], and a slower *in vivo* clearance of DA, which suggests lower basal DAT function in LEW rats [34]. These two strains also differ in several behaviors associated with DAergic neurotransmission including greater novelty-induced locomotor activity and vulnerability to drug self-administration in LEW rats compared to F344 rats (for reviews, see [35, 36]). These inbred strains thus provide a useful animal model of inherent variations in the integrity and function of the DA system, and any emerging behavioral differences between the F344 and LEW rats following exposure to radiation may be a function of differences in the DA systems of these strains.

The current study assessed the hypothesis that the severity of proton-induced rPVT deficits would be dependent on basal DAergic system differences, such that the F344 strain with its greater basal DA tone would display more severe radiation-induced rPVT deficits compared to the LEW strain. Given that the F344 rats have greater basal DA tone, it is possible that damage to this DAergic system will be more difficult to repair and/or require more resources in order to maintain stable rPVT performances following radiation exposure. This hypothesis is supported by work with humans demonstrating that DA alleles that confer greater DA tone in healthy humans are associated with a faster decline in PVT performance across the duration of the test session; that is, a greater decline in time-on-task and increased report of mental fatigue in participants with DA alleles associated with greater DA availability [37]. In the current study, F344 and LEW rats were trained to perform the rPVT and then exposed to head-only proton (25 or 100 cGy, 150 MeV) or sham irradiation. Post-irradiation rPVT performances were monitored for 34 weeks following exposure; levels of different dopamine proteins (tyrosine hydroxylase, dopamine transporter), cell-survival proteins (Akt, pAkt^ser473^, pCREB), or various cytokines were assessed in the frontal or parietal cortices of rats following the completion of the behavioral testing to determine if radiation differentially altered these proteins between the strains, since these brain areas are important for PVT performance in humans.

## Materials and Methods

### Subjects and Apparatus

Subjects were 17 male Fischer 344 rats (Harlan Laboratories, Indianapolis, Indiana) and 20 Lewis rats, acquired at approximately 10–12 weeks of age. Rats were housed in individual plastic cages and maintained on a 12:12 h light/dark cycle (lights on at 6:00 AM) and at an ambient temperature of 23°C for the duration of the experiment. Rats were run in 30-min rPVT sessions during their light-on cycle at the same time each day in identically constructed operant chambers. Each rPVT chamber contained one nose-poke key, cue lights, a house light, and a food cup for delivery of food pellets. All chambers were contained in sound-attenuating enclosures equipped with an exhaust fan. The weights of the rats were maintained at 85–90% of each strain’s free-feeding weight by feeding measured amounts of rat chow each day (30 min after the experimental session, 5 days/week; at similar times on the weekends), in addition to the food that was earned during the behavioral test sessions. Water was freely available in the home cage. For the rPVT procedure, experimental contingencies were controlled by MedPC® behavioral control programs running on PCs; the programs recorded all data on a trial-by-trial basis to provide for a wide range of subsequent analyses. Laboratory animal care was according to Public Health Service (PHS) Policy on the Humane Care and Use of Laboratory Animals. This study was carried out in strict accordance with the recommendations in the Guide for the Care and Use of Laboratory Animals of the National Institutes of Health. The protocol and all procedures were approved by the Institutional Animal Care and Use Committee of the Johns Hopkins University. Johns Hopkins also maintains accreditation of their program by the Association for the Assessment and Accreditation of Laboratory Animal Care (AAALAC).

### Rodent Psychomotor Vigilance Test (rPVT)

Rats were first trained to respond on a nose-poke key for food pellets on a fixed-ratio (FR) 1 schedule of reinforcement. Once this behavior was acquired, training on the rPVT procedure then began. Sessions began with the onset of the house light. After a variable delay of 3–10 sec the light behind the nose-poke key was illuminated. A correct response was defined as a response on the nose poke key within 1.5 sec after the light onset (i.e., 1.5-sec limited hold, LH) and was reinforced with a pellet. A response prior to the light onset (premature response) was not reinforced and punished with an 8 sec time out, while a response after the 1.5-sec interval had elapsed (miss) was not reinforced. The delay period for the next trial began after a 3-sec inter-trial interval, timed either after the response or the end of the 1.5-sec LH, whichever occurred first. Data collected were the numbers of correct responses as defined above, premature responses (responses that occurred prior to the light onset), misses (number of 1.5-sec light-on intervals during which no response occurred), and lapses in responding (misses plus responses greater than twice the rat’s mean response latency). Summary measures were expressed as percentages, as follows: accuracy = correct responses / (corrects + premature responses + misses); premature responding = number of premature responses / (corrects + premature responses + misses); lapse rate = (misses + lapses) / (corrects + premature responses + misses). In the human literature on PVT performance, lapses are considered an important indicator of inattention and/or fatigue and are typically defined as responses with latencies greater than 500 msec, or roughly twice the average latency for humans performing the 10-min version of the test. For rodents, average latency can vary considerably from subject to subject, and so the definition adopted here was based on each rat’s individual mean latency measure (described in detail in [32]). Premature responding was broken down further by calculating a false alarm (FA) rate for those premature responses occurring within the 3–10 second delay period (i.e., FA rate = premature responses within the 3–10 sec delay interval / (corrects + premature responses within the 3–10 sec delay interval + misses). The addition of a false alarm measure allowed for the calculation of a *d’* index of signal discriminability in which percent correct (PC) scores and false alarm (FA) rates are converted into *z* scores, and subtracted (d’ = z(PC)-z(FA); [38]).This performance measure is important for calculating a d prime (*d’*) measure of discriminability that can be compared across the different strains. Finally, response latencies to the light onset were recorded in milliseconds, and summarized by calculating both the median and mean reaction times.

The criterion for inclusion in the present study was that rats achieve at least 75% response accuracy and less than a 25% false alarm rate, resulting in a *d’* index of 1.35, for four out of the five daily test sessions during each of the two weeks prior to radiation. Most rats achieved this criterion more than two weeks prior to radiation, and the average pre-exposure *d’* index achieved for F344 and LEW rats was 1.9 and 2.0, respectively (accuracy of 78.1% and false alarm rate of 15.9% for F344; accuracy of 79.6% and false alarm rate of 15.9% for LEW). Thus all rats included in the study acquired and maintained stable rPVT performances prior to the exposure. In order to assign rats to radiation dose groups, a pseudo-random ranking technique was used for each strain. Specifically, a *d’* index was calculated for each rat across the five sessions completed during the week prior to radiation. Rats were ranked, within strain, from highest score to lowest score and were then assigned to one of the three dose groups (0, 25, and 100 cGy) such that the average *d’* index was comparable across groups (F344 and LEW sham: 2.1 ± 0.6 and 2.1 ± 0.3; F344 and LEW 25 cGy: 1.8 ± 0.2 and 2.1 ± 0.2; F344 and LEW 100 cGy: 1.9 ± 0.2 and 1.9 ± 0.2).

### Radiation Procedures

Rats were exported to Brookhaven National Laboratory (BNL) for radiation exposure 4–5 days prior to the scheduled exposure day. All animals were exposed on the same day, and then returned to Johns Hopkins for follow-up testing 4–5 days following exposure. While at BNL, rats were maintained under housing conditions similar to those at JHU, including the same light/dark cycle and were individually housed in plastic cages with a water bottle, wire top, and insulator top, that were filled with bedding and contained enrichment toys (i.e., Nyla bones). Rats also received *ad lib* access to water and food while at BNL. The control group was sham-irradiated, i.e., shipped to BNL, sedated and restrained for radiation exposure, but not actually exposed. The remaining rats were irradiated with 150 MeV/n protons generated at the NASA Space Radiation Laboratory (NSRL) facility at BNL. Protons of this energy have a mean range in water of approximately 15.5 cm with an average LET of 0.4 keV/μm. Target exposure levels were 25 and 100 cGy; actual delivered doses varied by no more than ±0.1 cGy relative to each target dose. Dose rates ranged from 30–71 cGy/min to achieve an even dose distribution across the collimated exposure field. Irradiation involved head-only exposures to minimize systemic responses to exposures that can confound morphological, neurochemical, and behavioral testing. For radiation exposures, rats received an intraperitoneal injection (2 ml/kg administered volume) of ketamine (90 mg/kg) and xylazine (5 mg/kg) and were subsequently placed in a ventilated polystyrene holder (2 rats/holder). Each body was shielded by a specially built trilaminar collimator exposure system consisting of Plexiglas^®^, aluminum, and polyurethane; the body dose was < 2% of the delivered dose.

### Protein Isolation from Brain Tissue

To determine the levels of various proteins in the brain following radiation exposure and behavioral testing, all rats were sacrificed at approximately 9-months (35–36 weeks) post-exposure. Whole rat brains were excised after decapitation, frontal and parietal cortices dissected, flash frozen, and immediately stored at -80°C until processed. Tissue sections were weighed and placed in a 1.5 ml centrifuge tube. Tissue was homogenized on ice using 3 ml of Radio Immuno Precipitation Assay (RIPA) buffer containing complete protease inhibitor and phophastase inhibitors per gram of tissue. Samples were then centrifuged at 12,000 x g for 10 min at 4°C, the pellet was discarded and the lysate transferred to a new microtube. Lysates were again centrifuged at 12,000 x g for 10 min at 4°C. The supernatant from the second centrifugation contains total protein lysate that was used for Western Blotting. Protein concentrations were calculated from each sample using Biorad Protein Assay Dye.

### Western Blotting to Detect Brain Proteins

Twenty μg of total protein from each rat were loaded onto 4–12% Bis-Tris gels and electrophoresed for 90 minutes at 110 volts in MOPS running buffer. Proteins were then electrophoretically transferred for 80 min at 30V onto PVDF membrane and subsequently incubated in Ponceau reagent for 10 minutes to ensure complete transfer of all proteins. After transfer, the membrane was blocked in 5% Non-fat dry milk in 1x Tris buffered saline containing 0.1% Tween 20 (TBST). Membranes were incubated with primary antibodies in TBST buffer for one hour at room temperature. The primary antibodies used were: Akt 1:1000; pAkt^ser473^ 1:1000; tyrosine hydroxylase (TOH) 1:500; pCREB 1:1000; dopamine transporter (DAT) 1:700; beta-actin, 1:2000. Membranes were washed (3 x 10 minutes) in TBST followed by incubation with secondary antibody at a dilution of 1:2500. After additional washing steps, West Dura chemilluminescence substrate was added to the membranes and they were allowed to incubate for 5 minutes. Membranes were visualized using a digital ChemiDoc-It imaging system. Densitometry was performed on all western blots and signal normalized to beta-actin, which serves as a housekeeping gene [39].

### Cytokine Array

An antibody-based cytokine array (Rat Cytokine Array Panel A, R&D Systems, Minneapolis, MN) was used to assess levels of 29 different cytokines in the frontal cortices of 25 cGy-exposed F344 and LEW rats and sham-irradiated controls of both strains. The 25 cGy rats were assessed with the array because it was at this radiation dose where the behavioral deficits were most severe in the F344 rats. The array procedure was carried out according to the manufacturer’s instructions.

### Data Analysis

For the rPVT, mean weekly performances were acquired for each rPVT behavioral measure for each rat, where five daily sessions per week were averaged to acquire each rat’s mean weekly performance for percent corrects (i.e., accuracy), false alarm rates, lapses, and median reaction times. Each behavioral measure was analyzed with separate repeated-measures ANOVAs, with Week as the repeated-factor and Strain and Radiation Dose as the between-subjects factors. Specific group differences were assessed with Tukey-corrected or Dunnett’s test as post-hocs. In the case of a significant Mauchly’s sphericity test, a Greenhouse-Geisser correction was applied to control Type I error; Greenhouse-Geisser revised degrees of freedom are provided below. Alpha was set to p = 0.05. All statistical calculations were performed using SPSS Statistics (v20; IBM Corporation).

For the protein analyses, densitometry was calculated, and ratios of each protein/beta actin were calculated for individual rats in each proton dose level group. These protein/actin ratios were compared between sham-irradiated controls of each strain with independent-samples t-tests to determine if strain differences were apparent. To enable between strain comparisons in the event of strain difference between sham controls, rats’ protein/actin ratios were normalized to their strain-specific sham-irradiated control group, which were both set to 1. Each protein was then analyzed using two-way ANOVAs with Strain and Radiation Dose as the between-subjects factors. Specific group differences were assessed with Tukey-corrected or Dunnett’s test as post-hoc tests. All statistical calculations were performed using SPSS Statistics.

For the rat cytokine array, duplicate samples were averaged and converted to a percent of control value for each strain. Control data was set to 1. Independent-samples t-tests were used to assess within-strain differences between sham controls and 25 cGy irradiated rats.

## Results

### rPVT Behavioral Performances

**Fig 1:**
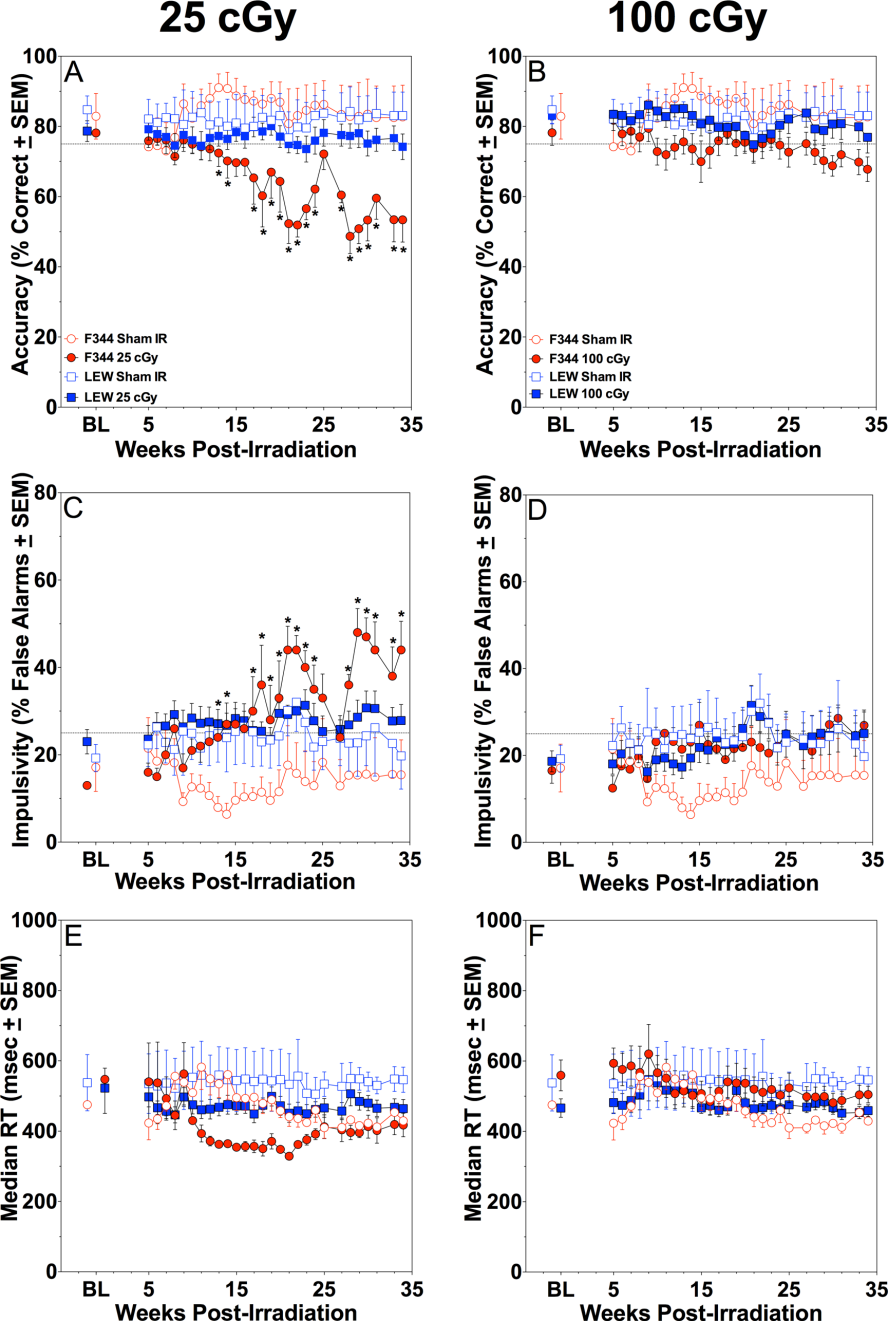
Proton Radiation Results in rPVT Deficits in F344 Rats. Mean rPVT performance across 34 weeks post-irradiation in F344 and LEW rats (n = 4–8 rats/group). Accuracy (percent correct) for 25 cGy-exposed (A) or 100 cGy-exposed (B) F344 and LEW rats; sham controls appear on all panels. Impulsivity (false alarm rate) for 25 cGy-exposed (C) or 100 cGY-exposed (D) F344 and LEW rats; median reaction time for 25 cGy-exposed (E) or 100 cGy-exposed (F) F344 or LEW rats. *denotes significant difference from F344 sham control, other details appear in the text. BL = baseline rPVT performance for each group the week prior to irradiation.

For percent correct scores, a repeated-measures ANOVA with a Greenhouse-Geisser correction revealed a significant within-subjects main-effect of Week [F(3.293, 102.078) = 3.078, p = 0.027] and a significant within-subjects interaction of Week X Strain X Radiation Dose [F(6.586, 102.078) = 2.246, p = 0.04]; significant between-subjects main-effect of Radiation Dose [F(2, 31) = 5.357, p = 0.01] was also found. No other significant effects or interactions were apparent (all p’s ≥ 0.063). Tukey-corrected post-hocs showed a significant decrease in percent correct scores for the 25 cGy-exposed F344 rats at Weeks 13, 14, 17–24, 28–31, and 34 post-irradiation when compared to F344 and LEW sham controls, and Week 33 for the F344 sham controls (Fig 1). The 25 cGy-exposed F344 rats differed from all other groups on Weeks 22 and 29. They also differed from both LEW exposed groups on Weeks 18, 30 and 34 and from LEW 100 cGy-exposed rats on Weeks 23 and 31.

While not a statistically significant decrease, the 100 cGy-exposed F344 rats did show a subtle decrease over time in percent correct scores, with a weekly mean of 81% correct immediately following radiation exposure to a weekly mean of 67% correct at 34 weeks post-exposure. Importantly, this group of rats was consistently below the 75% correct criterion starting at Week 28 post-irradiation, suggesting that these rats displayed a less-severe performance deficit compared to the 25 cGy-exposed rats. In comparison, both exposed groups of LEW rats maintained percent correct scores at or above 75% correct throughout the post-exposure period.

For false alarm rates, a repeated-measures ANOVA with a Greenhouse-Geisser correction determined a significant within-subject main-effect of Week [F(3.516, 109.011) = 5.256, p = 0.001] and a significant within-subjects Week X Strain X Radiation Dose interaction [F(7.033, 109.011) = 2.393, p = 0.026]; a significant between-subjects main-effect of Radiation Dose [F(5.984, 31) = 5.984, p = 0.006] was also found. No other main-effects or interactions were significant (all p’s ≥ 0.077). Tukey-corrected post-hocs showed a significant increase in false alarm rates in the 25 cGy-exposed F344 rats at Weeks 13, 14, 17–24, 28–31, 33, and 34 compared to F344 and LEW sham controls (Fig 1). The 25 cGy-exposed F344 rats differed from all other exposed groups on Weeks 22, 23, and 29. They also differed from both LEW exposed groups on Weeks 18, 30 and 34 and from LEW 100 cGy-exposed rats on Week 31.

For median reaction time, a repeated-measures ANOVA with a Greenhouse-Geisser correction revealed only a significant within-subjects main-effect of Week [F(4.007, 124.220) = 3.918, p = 0.005); no other significant main-effects or interactions were found (all p’s ≥ 0.068; see Fig 1, bottom panels). Similar results were found for lapses, where a repeated-measures ANOVA with a Greenhouse-Geisser correction revealed a significant within-subjects main-effect of Week [F(5.997, 185.910) = 6.100, p < 0.001] and a significant between-subjects main-effect of Strain [F(1, 31) = 6.043, p = 0.02]; no other significant main-effects or interactions were found (all p’s ≥ 0.081; data not shown).

### Frontal Cortex Protein Analyses

**Fig 2:**
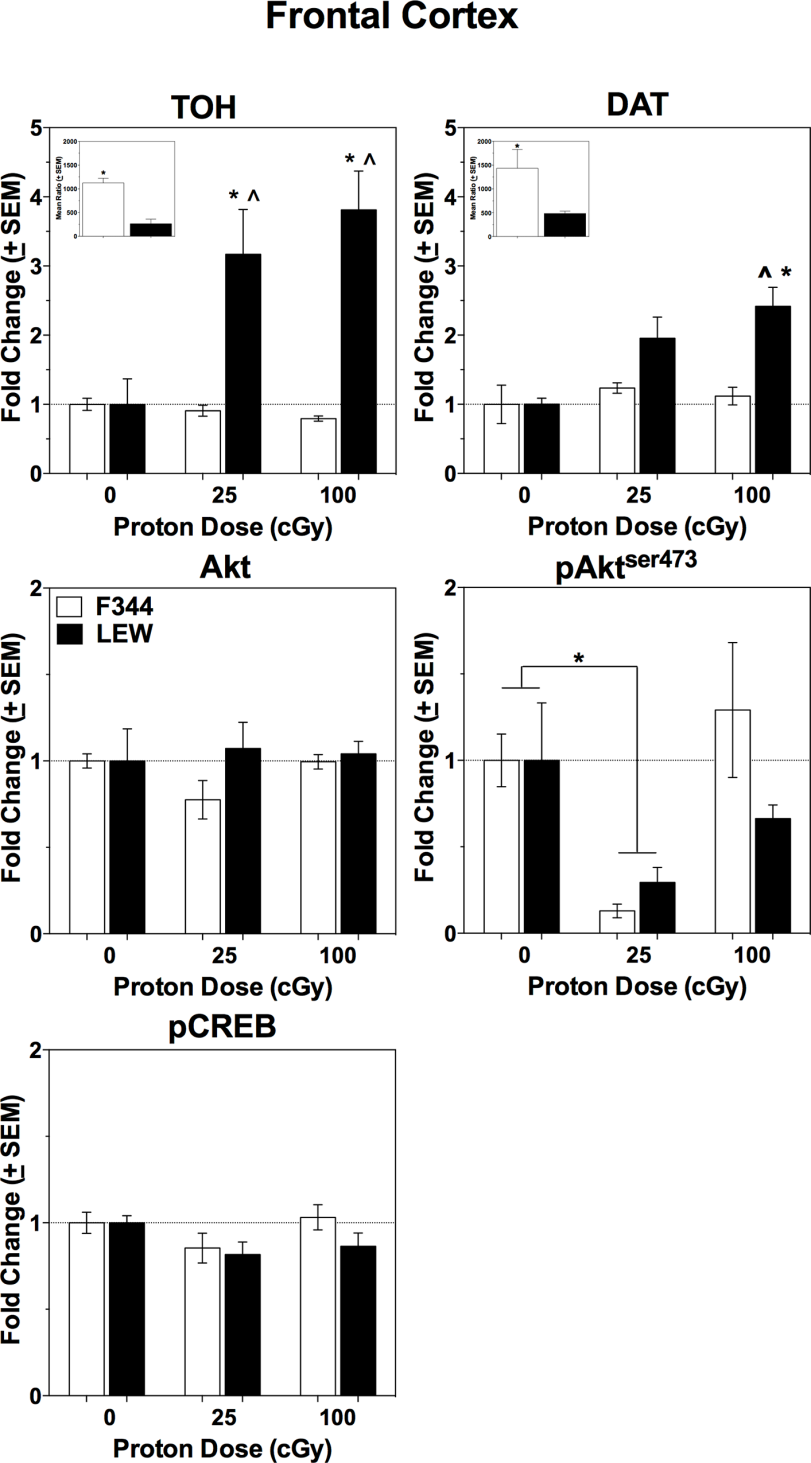
Frontal Cortex Protein Expression in Proton-Exposed F344 and LEW Rats. Dopamine and cell-survival related protein expression in the frontal cortex of F344 and LEW rats (n = 4–8 rats/group) at 35 weeks post-IR, after completion of the rPVT behavioral testing period. *denotes significant difference from sham controls. ^denotes significant between-strain difference at that proton dose. Insets: mean ratio of protein/beta-actin to demonstrate previously reported strain difference in dopamine-related protein levels; *denotes greater TOH (left inset) and DAT (right inset) protein levels in F344 sham controls compared to LEW sham controls.

Five different proteins were examined in the frontal cortices of all F344 and LEW rats: tyrosine hydroxylase (TOH), the rate-limiting enzyme for dopamine synthesis; the dopamine transporter (DAT); Akt and phospho-Akt (ser473; pAkt^ser473^); and phospho-CREB (pCREB). Strain differences in the levels of TOH and DAT in the frontal cortices of these rats replicated previous research [33, 34, 40], with greater TOH and DAT levels in F344 rats compared to LEW rats (independent-samples t-tests, TOH p = 0.001 and DAT p = 0.019; see insets on Fig 2 for TOH and DAT). No other strain differences were apparent between the sham-irradiated controls. There were no significant differences in levels of Akt and pCREB following radiation in the F344 and LEW rats (all p’s ≥ 0.174; Fig 2). For TOH, the ANOVA revealed significant effects of Strain [F(1, 36) = 20.530, p < 0.05], Radiation Dose [F(2, 36) = 3.920, p = 0.03], and a significant Strain X Radiation Dose interaction [F(2, 36) = 5.198, p = 0.011]. LEW rats exposed to 25 cGy or 100 cGy displayed a similar increase in TOH in the frontal cortex that was significantly different from TOH levels in F344 rats exposed to either proton dose and sham controls of both strains. DAT levels were also significantly altered by radiation, with the ANOVA revealing a significant effect of Strain [F(1, 36) = 9.914, p = 0.004], Radiation Dose [F(2, 36) = 4.479, p = 0.02], and Strain X Radiation Dose interaction [F(2, 36) = 3.237, p = 0.05]. Following radiation, 100 cGy-exposed LEW rats displayed greater frontal cortex DAT levels compared to all other groups (p = ≤ 0.035), except compared to LEW rats exposed to 25 cGy (p = 0.684). For pAkt^ser473^, the ANOVA revealed a significant main effect of Radiation Dose [F(2, 36) = 4.458, p = 0.020]; the effect of Strain and Strain X Radiation Dose interaction were not significant (all p’s ≥ 0.359). Levels of this protein were significantly decreased in 25 cGy-exposed rats, regardless of strain, when compared to sham-irradiated controls and 100 cGy exposed rats (all p’s < 0.05).

### Parietal Cortex Protein Analyses

**Fig 3:**
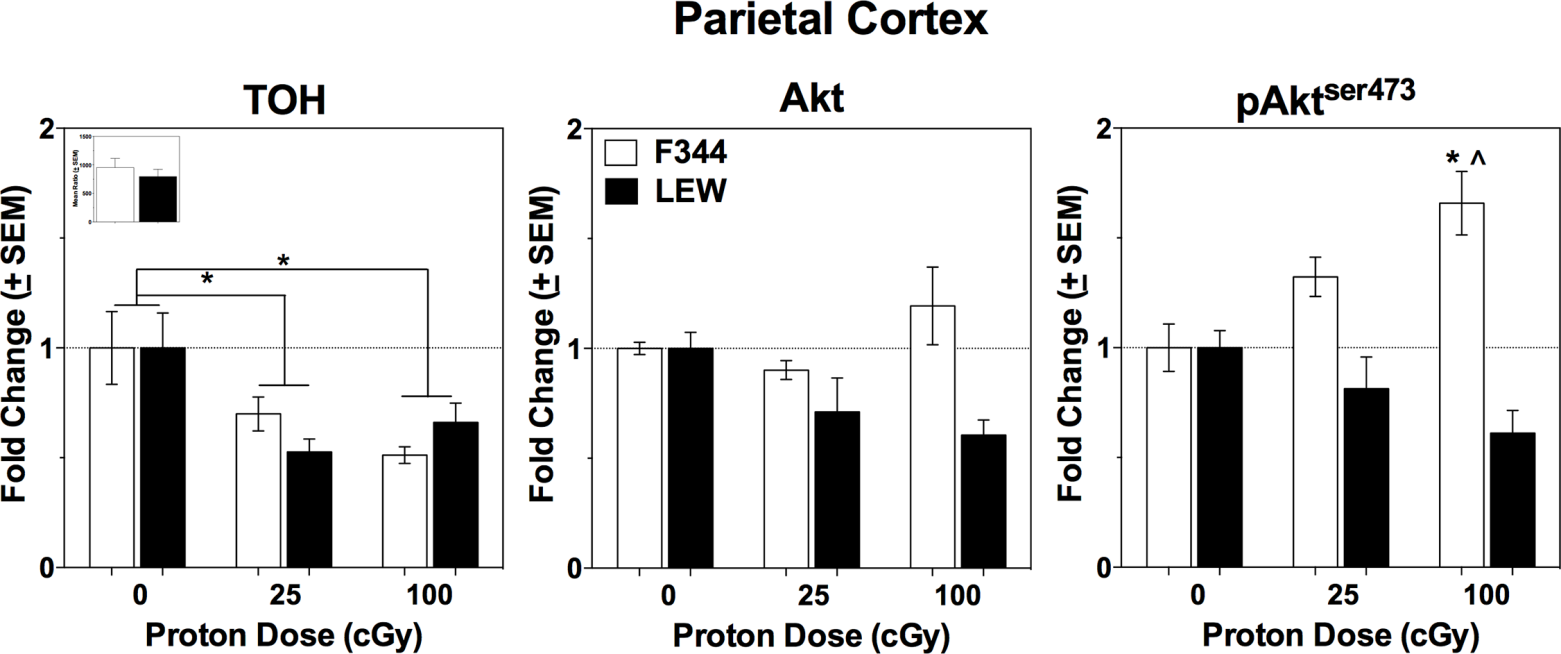
Parietal Cortex Protein Expression in Proton-Exposed F344 and LEW Rats. Dopamine and cell-survival related protein expression in the parietal cortex of F344 and LEW rats (n = 4–8 rats/group) at 35 weeks post-IR, after completion of the rPVT behavioral testing period. *denotes significant difference from sham controls. ^denotes significant between-strain difference at that proton dose. Inset: mean ratio of TOH/beta-actin to demonstrate no strain difference in dopamine-related protein levels in the parietal cortex.

**Fig 4:**
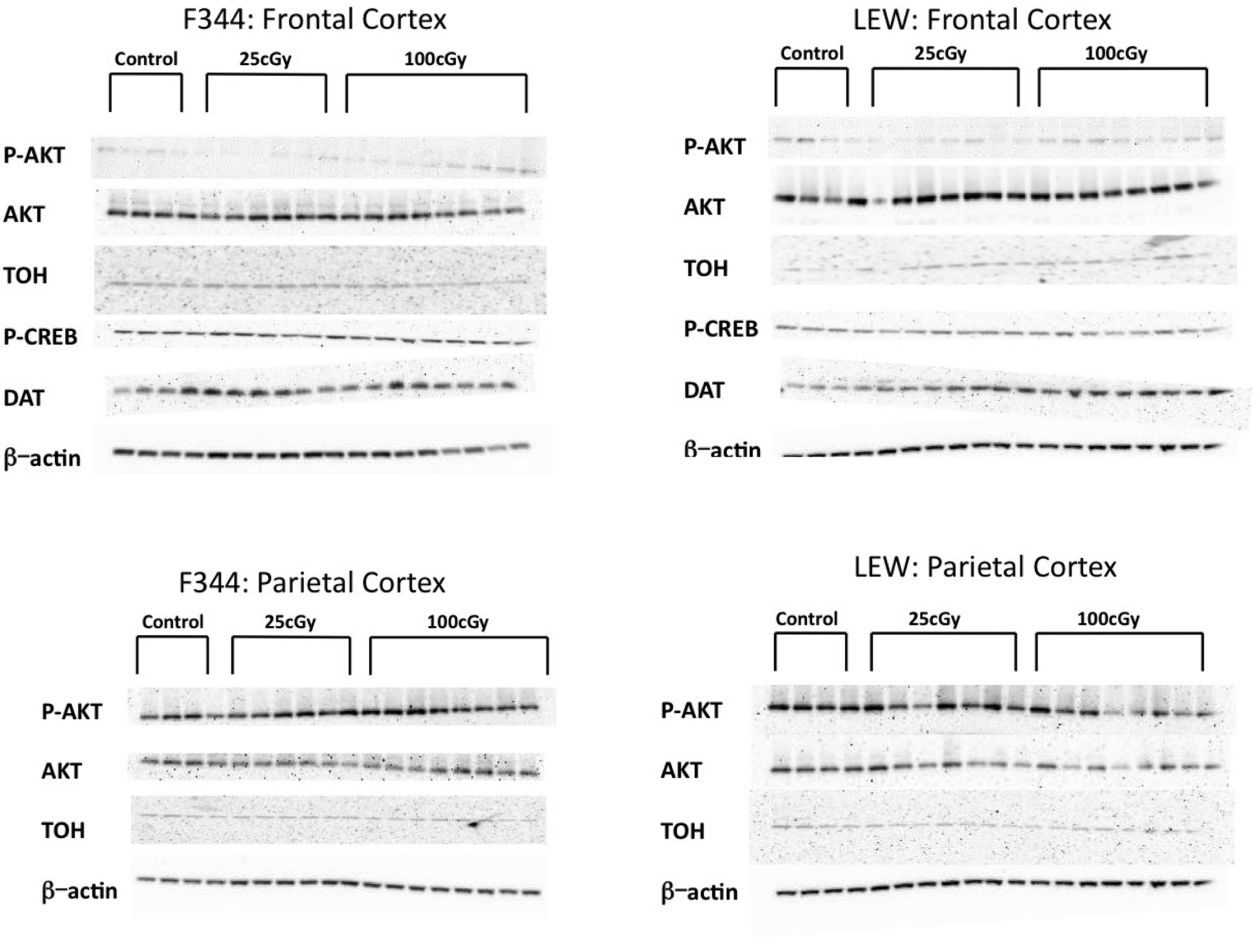
Western Blots for Frontal and Parietal Cortex Protein Expression Assays. Western blots from F344 and LEW rats frontal (top panels, left and right, respectively) and parietal (bottom panels, left and right respectively) cortices.

Three different proteins were examined in the parietal cortices of all F344 and LEW rats: TOH, Akt, and pAkt^ser473^. For TOH, the ANOVA revealed a significant effect of Radiation Dose [F(2, 36) = 11.673, p < 0.05], but not Strain (p = 0.913) or the Strain X Radiation Dose interaction (p = 0.162). TOH was significantly decreased in the parietal cortex of all irradiated rats, regardless of proton dose, when compared to sham-irradiated controls (all p’s ≤ 0.001; Figs 3 and 4). TOH levels did not, however, differ between proton dose groups (p = 0.989). For Akt, the ANOVA revealed no significant effects of Radiation Dose (p = 0.542) or Strain X Radiation Dose interaction (p = 0.141), although the effect of Strain trended towards significance (p = 0.054). For pAkt^ser473^, the ANOVA revealed a significant effect of Strain [F(1, 36) = 18.693, p < 0.05] and Strain X Radiation Dose interaction [F(2, 36) = 6.880, p = 0.003], but no main-effect of Radiation Dose (p = 0.641). On average, pAkt^ser473^ decreased with increasing radiation dose in the LEW rats and increased with increasing radiation dose in the F344 rats. These changes were significantly different in the rats exposed to 100 cGy, with F344 rats displaying significantly more of this protein compared to sham-irradiated controls and both LEW proton-exposed groups (p ≤ 0.029), but not F344 rats exposed to 25 cGy (p = 0.563). LEW rats exposed to 100 cGy protons displayed a significant decrease in this protein compared to both 25 and 100 cGy-exposed F344 rats (all p’s ≤ 0.02), but were not different from sham-irradiated controls of either strain or LEW rats exposed to 25 cGy (all p’s ≥ 0.441).

### Cytokine Array Analyses

Twenty-nine different cytokines were assessed with the array in the frontal cortex of F344 and LEW rats and are listed in Table 1. For the 25 cGy-exposed F344 rats, no cytokines were significantly elevated compared to F344 sham-controls, but several cytokines were significantly decreased, including Regulated on Activation, Normal T Cell Expressed and Secreted (RANTES), interleukin-6 (IL-6), CXCL9 (MIG), and CXCL7 (Thymus Chemokine; all p’s ≤ 0.05). Additionally, decreases in granulocyte macrophage colony-stimulating factor (GM-CSF; p = 0.07), fractalkine (CX3CL1; p = 0.09), soluble intracellular adhesion molecule-1 (sICAM-1; p = 0.086), vascular endothelial growth factor (VEGF; p = 0.07), and interferon-γ (IFN-γ; p = 0.07) approached significance. For the LEW rats, several cytokines were significantly elevated compared to LEW sham controls, including ciliary neurotrophic factor (CNTF), GM-CSF, sICAM-1, and fractlkine (CX3CL1). Cytokines that were significantly decreased in this group include interleukin-13 (IL-13), LIX (CXCL5), L-Selectin (CD62L), and macrophage inflammatory protein-1α (MIP-1α or CCL3). In support of the LEW and F344 rats’ known genetic differences in hypothalamic-pituitary-adrenal axis activity, inflammatory responses, and susceptibility to various infections, autoimmune disorders, and tumors [41–43], the low corticosterone/high pro-inflammatory irradiated LEW rats displayed a 3.18 fold increase in IFN-γ, whereas the high corticosterone/low pro-inflammatory irradiated F344 rats displayed a 40% decrease in IFN-γ that approached significance (p = 0.07).

**Table 1:** Expression Level of Frontal Cortex Cytokines From 25 cGy Proton-Irradiated F344 and LEW Rats.

Cytokines	F344	LEW
TNF-α	-	118 ± 73
IL-1α	70 ± 25	30 ± 42
IL-1β	82 ± 15	100 ± 55
IL-1ra	115 ± 2	70 ± 57
IL-2	94 ± 36	137 ± 94
IL-3	72 ± 32	78 ± 20
IL-4	61 ± 17	118 ± 13
IL-6	21 ± 10*	122 ± 53
IL-10	62 ± 24	97 ± 19
IL-13	31 ± 22	56 ± 3*
IL-17	53 ± 2	35 ± 31
IP-10	341 ± 54	52 ± 17^
LIX	97 ± 1	39 ± 3*
L-Selectin	51 ± 21	67 ± 5*
IFN-γ	60 ± 17^	318 ± 113
CINC-1	73 ± 38	10 ± 15
CINC-2α/β	110 ± 50	-
CINC-3	138 ± 112	158 ± 72
CNTF	106 ± 1	206 ± 10*
Fractalkine	56 ± 11^	228 ± 23*
GM-CSF	22 ± 28^	236 ± 27*
sICAM-1	74 ± 7^	242 ± 48*
MIG	41 ± 16*	114 ± 32
MIP-1α	22 ± 7	50 ± 0.6*
MIP-3α	48 ± 23	49 ± 2
RANTES	5 ± 2*	32 ± 7
Thymus Chemokine (CXCL7)	90 ± 3*	75 ± 7^
TIMP-1	77 ± 23	41 ± 30
VEGF	45 ± 16^	83 ± 33

Results are expressed as the percentage of each frontal cortex cytokine expressed in each strain’s sham-irradiated control group. Results are mean ± SD.

* Significantly different from within-strain sham-irradiated control group (p ≤ 0.05).

^ Trend (p = 0.06–0.09) when compared to within-strain sham-irradiated control group.

## Discussion

The results indicate that F344 rats exposed to head-only protons at 25 cGy show significant decreases in accuracy and significant increases in impulsive responding, while LEW rats do not. Importantly, 100 cGy-exposed F344 rats did display a decrease in rPVT performance over the post-exposure period, even though this difference was not significantly different from F344 sham controls. For example, the 100 cGy-exposed F344 rats displayed a more subtle decrease in accuracy compared to the 25 cGy-exposed F344 rats, and the 100 cGy-exposed F344 rats were consistently below the 75% correct criterion from starting at Week 28. While this change was not significantly different from the F344 sham controls, performance consistently below the 75% correct criterion could be considered a performance deficit. In comparison, both of the irradiated LEW groups maintained accuracy values at or above 75% correct for the duration of the post-exposure period.

These behavioral deficits (or lack thereof) were accompanied by strain-specific changes in various proteins in the frontal and parietal cortices, two brain areas important for sustained attention and performance of the PVT in humans. Specifically, radiation exposure decreased TOH in the parietal cortex in all irradiated groups, but only the LEW rats displayed a concomitant *increase* in TOH levels in the frontal cortex. This increase in frontal cortex TOH in LEW rats was also accompanied by an increase in the DAT protein. No changes in frontal cortex DAT levels were apparent in the irradiated F344 rats. Thus, radiation exposure induced brain protein changes in a strain-specific manner, suggesting that the LEW rats were able to adapt to or mitigate the radiation-induced damage, which resulted in a lack of behavioral deficits following head-only proton exposure.

In a previous report from our laboratory, proton-induced rPVT deficits including decreased accuracy and increased impulsive responding, were associated with increased whole-brain DAT protein levels in 100 cGy-exposed, but not 25 cGy-exposed outbred Long-Evans rats. Further, these behavioral and neurochemical changes were evident in approximately 40% of the exposed cohort, which suggests the presence of individual differences in sensitivity to the deleterious effects of proton radiation on neurobehavioral performance. On the surface, the data in the current report appear to contradict these previous results, given that the LEW rats displayed increased DAT levels, but did not display radiation-induced deficits in rPVT performance throughout the 34-week post-IR testing period; however, the differences in basal dopaminergic tone between these inbred strains could underlie these results and have important implications for interpretation of our previous report in light of pre-irradiation differences in the dopaminergic neurotransmitter system.

First and most importantly, it is currently unknown whether increased DAT protein levels in whole brain or the frontal cortex are solely a function of radiation exposure or are a pre-existing neurochemical difference that impacts an organism’s behavioral responses to radiation exposure. Indeed, the F344 and LEW rats display differences in dopaminergic protein levels, including DAT, and *in vivo* DAT activity in various brain regions [33, 34], and utilizing these known strain differences as a model to test the importance of pre-existing differences in the dopamine system on radiation-induced neurobehavioral deficits was the primary goal of the current study. Thus, the F344 rats, a strain with greater dopaminergic tone and protein levels, were hypothesized to have more severe radiation-induced deficits following proton exposure compared to the LEW strain, a hypothesis supported by the results of the current study. These deficits were apparent even though the F344 rats showed no radiation-induced increase in DAT levels compared to F344 sham controls following radiation exposure. While these results appear contradictory, the fact that 1) the F344 sham controls had *greater* frontal cortex DAT levels than the LEW sham controls prior to adjusting all data to a change from each strain’s sham control values (see Fig 2, inset on DAT panel for mean DAT/beta-actin ratios in F344 and LEW shams) and 2) both irradiated F344 groups had DAT levels equivalent to the F344 sham controls, supports the hypothesis that greater DAT protein levels (and possibly DA tone) are associated with poorer neurobehavioral performance following radiation exposure. In addition, this same strain difference was evident in TOH levels in the frontal cortex (see Fig 2, inset on TOH panel), but not in TOH levels in the parietal cortex (see Fig 3, inset on TOH panel), which suggests that these strain differences are specific to dopamine availability in the frontal cortex.

Second, it is likely that whole-brain DAT protein levels at the end of the post-IR testing period are not always reflective of brain region-specific DAT protein levels at that time or at different time points following radiation, such that the “radiation sensitive” Long Evans rats in our prior report could have had differential DAT levels in certain brain regions at different times post-IR, and yet still displayed greater overall whole brain DAT protein levels after approximately 40 weeks post-IR. Whole-brain DAT levels could be higher in the F344 rats compared to LEW rats, but only brain region-specific assessments of proteins were completed at the conclusion of the post-IR period in the current study. While the protein data are important with regard to the possible brain changes following radiation exposure, it remains unknown if DA levels or levels of its metabolites are differentially altered in rats with neurobehavioral deficits in the rPVT and how these levels might change throughout the post-IR testing period. Recently, a decrease in frontal DA and norepinephrine levels was reported 24-hours after exposure to 100 cGy protons (170 MeV), a dose and energy similar to those used in the current study, in addition to deficits in the conditioned passive avoidance task [26]. Thus, DA and other monoamines could be immediately decreased following proton radiation and the response to this change in monoamine levels could predict the severity of cognitive deficits following radiation exposure. To better address these questions, additional studies are needed that quantify monoamine receptor and transporter protein levels, in addition to the monoamines themselves and their metabolites, in specific brain regions and whole brain immediately following radiation and periodically across the post-IR period.

As expected, the pattern of cytokine changes in the frontal cortex was strain-specific. For example, the standard proinflammatory cytokines such as IL-1b, IL-6, and TNF-α were not consistently elevated in either strain; IL-6 was significantly decreased in the 25 cGy-exposed F344 rats, but unchanged in the irradiated LEW rats. Interestingly, several cytokines that are considered neurotrophic and/or important for cognitive function were differentially altered in the F344 and LEW rats and could provide promising potential therapeutic leads in future studies. In particular, CNTF was two-fold higher in 25 cGy-exposed LEW rats compared to the LEW sham controls. This cytokine could be important for the maintenance of control-like rPVT performance in this group, given that administration of CNTF reportedly improves cognitive function and/or motor performance in preclinical models of Alzheimer’s disease, Huntington’s disease, Parkinson’s disease, and amyotropic lateral sclerosis (for a review, see [44]). Further, 25 cGy LEW rats displayed a 2.5 fold increase in GM-CSF levels (vs. LEW sham controls), compared to a 75% reduction (that approached significance) in this cytokine in 25 cGy F344 rats. Since patients with chronic fatigue syndrome have lower GM-CSF levels in cerebrospinal fluid [45], these results suggest that this cytokine could be an important mediator of “fatigue-like” effects in preclinical models. Since the PVT was designed to detect fatigue-induced decreases in human performance, more work is needed to determine if fatigue can be detected in this rodent model. Finally, the neurohormonal phenotypes evident in the F344 and LEW rats are commonly used to study genetic differences to various immunologic challenges. Importantly, neurohormonal phenotypes resembling the F344 and LEW rats occur in humans and are dependent upon baseline epinephrine and norepinephrine levels, two classical monoamines [41]. While the current study was designed to assess dopamine specifically, the importance of other monoamines in an organism’s response to radiation requires further study because monoamine-mediated differences in baseline inflammatory responses could be important for how individuals respond to and repair radiation-induced injury.

Taken together, the current results demonstrate that baseline dopaminergic tone is an important factor in response to radiation exposure, with more severe deficits evident in rats with greater DA tone and protein levels. Further, trophic cytokines could play an important role in retaining normal cognitive performance following radiation exposure. More work is needed to determine how monoamine neurotransmission is altered by radiation exposure, how these changes relate to behavioral deficits, and if trophic factors have therapeutic potential for mitigating or eliminating radiation-induced cognitive deficits in clinical populations.

## Supporting Information

S1 Table Mean weekly rPVT accuracy (percent correct) for F344 and LEW rats. (PDF)

S2 Table Mean weekly rPVT false alarms (impulsivity) for F344 and LEW rats. (PDF)

S3 Table Mean weekly rPVT median reaction time for F344 and LEW rats. (PDF)

S4 Table Mean protein fold change in frontal and parietal cortices of F344 and LEW rats. (PDF)
