# Supplementary material for: Deficits in Sustained Attention and Changes in Dopaminergic Protein Levels following Exposure to Proton Radiation Are Related to Basal Dopaminergic Function
Source: PLoS One. 2015 Dec 10;10(12):e0144556. doi: 10.1371/journal.pone.0144556 (PMC4684339; doi:10.1371/journal.pone.0144556)
Supplement: S1 Table — (PDF) [file pone.0144556.s001.pdf]

Percent Correct Week	F344 Sham		F344 25 cGy		F344 100 cGy		LEW Sham		LEW 25 cGy		LEW 100 cGy	
	Mean	SEM	Mean	SEM	Mean	SEM	Mean	SEM	Mean	SEM	Mean	SEM
-1	82.89	6.51	78.15	1.08	78.21	3.59	84.77	3.92	78.67	2.96	83.06	2.34
0												
1												
2												
3												
4												
5	74.19	8.18	75.90	1.93	81.75	2.90	82.12	5.60	79.19	3.11	83.45	2.55
6	74.53	7.91	76.55	1.82	77.88	3.37	81.12	5.29	77.82	3.19	83.16	3.53
7	73.04	8.07	76.13	3.87	78.62	3.38	82.53	4.05	76.99	2.63	81.61	3.84
8	76.86	6.12	71.42	2.35	77.04	4.28	82.21	6.10	74.54	3.38	83.33	3.51
9	86.42	5.67	76.14	3.25	79.45	3.75	80.55	9.85	77.55	3.54	86.07	2.15
10	82.36	5.70	74.92	2.52	72.83	3.83	82.61	7.33	76.06	3.80	84.41	2.56
11	85.91	4.74	74.16	3.60	71.95	4.39	83.91	5.12	74.49	4.01	82.79	3.04
12	87.92	4.35	73.60	3.36	74.06	3.36	80.45	6.78	76.71	3.40	85.01	2.30
13	91.05	3.91	72.42	3.83	75.61	3.47	81.28	5.74	77.28	2.89	85.1	2.74
14	90.78	4.61	70.16	4.89	73.58	4.36	80.07	7.68	76.46	3.02	83.12	2.72
15	88.68	5.03	69.67	3.92	69.97	5.91	80.97	8.18	78.36	2.62	80.72	3.42
16	87.66	3.81	69.78	3.77	73.09	4.99	79.53	9.78	77.2	3.38	81.77	2.98
17	87.24	2.84	65.34	7.49	75.93	4.20	80.02	7.62	79.83	2.79	79.72	3.75
18	86.36	4.34	60.24	8.88	77.93	3.04	82.59	6.25	78.56	2.38	79.74	3.14
19	87.97	4.76	66.99	7.42	75.17	3.51	81.77	6.35	80.1	2.60	80.02	4.50
20	86.94	5.75	64.33	8.75	75.51	3.18	83.09	6.30	77.09	3.12	77.42	4.49
21	80.80	9.90	52.30	5.63	73.71	4.17	76.73	7.43	74.99	2.50	74.79	4.71
22	83.08	7.79	51.91	3.41	75.03	4.17	79.87	7.69	74.69	2.40	76.58	3.77
23	84.58	7.25	56.59	3.16	76.29	3.09	79.57	7.26	73.56	3.68	77.85	3.75
24	86.05	6.22	62.14	5.17	74.68	3.97	83.11	5.67	75.96	3.85	80.35	3.91
25	86.26	6.77	72.15	4.45	72.67	4.24	83.76	6.50	78.18	3.78	82.13	4.14
26												
27	83.38	8.35	60.44	2.19	75.09	3.26	82.6	6.97	77.53	3.14	83.82	4.99
28	82.87	8.64	48.69	4.92	72.61	3.43	84.22	7.05	77.28	3.46	79.29	4.57
29	82.86	9.66	50.87	4.27	70.22	3.51	81.89	6.64	78.05	4.10	78.85	4.32
30	83.48	10.00	53.33	5.95	68.77	2.94	82.59	6.12	75.12	3.54	80.68	4.04
31	82.53	8.99	59.56	6.01	71.95	3.48	83.64	7.23	76.21	3.28	80.8	4.50
32												
33	82.53	8.99	53.42	6.32	69.83	3.59	83.08	6.71	76.66	3.49	79.91	4.20
34	82.73	9.02	53.40	6.35	67.80	3.50	83.08	6.71	74.2	3.63	76.9	4.49
