# Supplementary material for: Deficits in Sustained Attention and Changes in Dopaminergic Protein Levels following Exposure to Proton Radiation Are Related to Basal Dopaminergic Function
Source: PLoS One. 2015 Dec 10;10(12):e0144556. doi: 10.1371/journal.pone.0144556 (PMC4684339; doi:10.1371/journal.pone.0144556)
Supplement: S3 Table — (PDF) [file pone.0144556.s003.pdf]

Median RT Week	F344 Sham		F344 25 cGy		F344 100 cGy		LEW Sham		LEW 25 cGy		LEW 100 cGy	
	Mean	SEM	Mean	SEM	Mean	SEM	Mean	SEM	Mean	SEM	Mean	SEM
-1	475.33	9.58	522.40	96.90	559.36	43.86	537.87	69.16	522.42	56.64	466.35	26.16
0												
1												
2												
3												
4												
5	423.33	47.92	540.6	110.30	593.75	43.15	535.32	84.80	497.51	28.41	481.94	29.80
6	434.67	29.59	537.39	116.06	576.04	46.08	528.72	98.44	466.63	19.20	474.17	33.23
7	471.83	14.58	492.88	61.54	586.01	56.98	537.01	93.61	456.93	20.78	487.57	34.17
8	556	36.38	445.2	40.70	567.5	74.67	522.17	85.07	446.44	22.73	503.06	41.72
9	539.28	5.17	563.1	88.73	620.34	83.90	544.58	82.95	497.18	27.05	547.26	60.54
10	509.33	7.16	430.34	8.16	566.68	48.92	553.47	85.51	475.12	25.84	537.93	51.27
11	582.25	63.76	393.38	24.02	551	54.44	559.5	96.26	460.76	24.75	517.25	48.38
12	548.85	45.69	372	13.60	507.43	42.64	538.53	78.03	462.74	27.92	524.11	48.31
13	534.65	38.99	362.79	10.00	515.03	45.46	559.8	80.20	468.02	24.56	519.68	39.57
14	560.9	69.94	364.34	9.52	502.53	37.15	546.47	89.87	475.47	26.84	508.95	38.55
15	495.28	36.20	354.48	3.70	507.45	35.44	543.87	88.35	471.84	23.47	466.93	15.60
16	493.03	33.35	356.6	55.64	485.41	39.34	548.33	89.04	471.53	20.83	473.95	26.38
17	496.45	35.23	356.8	62.19	514	43.97	541.47	85.61	448.93	24.73	460.27	18.70
18	478.75	28.71	350.13	63.19	540.96	44.68	548.2	87.75	473.96	20.55	469.86	23.45
19	489.45	31.88	371.07	21.56	537.68	56.01	543	86.48	498.78	27.06	516.43	27.38
20	458	19.39	348.33	7.92	537.05	42.19	546.73	86.33	472.31	26.81	480.88	28.41
21	441.2	27.75	328.73	5.79	519.93	34.81	533.47	87.05	450.99	25.84	464.2	22.25
22	436.16	24.59	362.15	7.33	511.07	38.66	556.75	104.04	457.74	24.61	467.8	17.40
23	424.75	8.81	375.73	15.18	519.05	31.25	507.73	57.38	449.47	26.42	474.21	16.05
24	460	34.81	391.53	15.37	503	28.76	505.27	47.48	461.78	21.91	464.53	15.19
25	410	30.01	412.67	33.61	523.75	42.16	533.83	34.92	466.17	26.54	475.56	24.67
26												
27	410.45	14.90	403.87	33.28	498.6	28.83	527.41	65.58	457.23	24.39	469.26	15.98
28	432.25	9.01	396.27	20.32	497.7	28.07	549.8	45.49	506.42	34.08	479.68	18.19
29	415.85	23.31	395.67	13.69	499.03	28.96	548.87	32.15	484.78	24.89	484.75	22.51
30	422.81	9.37	413	32.68	481.28	27.42	540.92	27.14	479.82	21.48	467.41	18.52
31	411.96	16.78	401.89	36.04	487.73	26.16	530.17	23.79	465.26	23.04	452	18.18
32												
33	453.5	8.52	419.07	29.23	503.98	27.10	548.33	35.22	467.69	24.20	453.54	17.43
34	429.55	9.22	417.55	33.46	504.73	23.66	546.33	35.66	464.08	25.89	459.2	16.37
