# Supplementary material for: Deficits in Sustained Attention and Changes in Dopaminergic Protein Levels following Exposure to Proton Radiation Are Related to Basal Dopaminergic Function
Source: PLoS One. 2015 Dec 10;10(12):e0144556. doi: 10.1371/journal.pone.0144556 (PMC4684339; doi:10.1371/journal.pone.0144556)
Supplement: S4 Table — (PDF) [file pone.0144556.s004.pdf]

Frontal	F344-TOHFC		LEW-TOHFC		F344-pCREBFC		LEW-pCREBFC		F344-AktFC		LEW-AktFC	
	Mean	SEM	Mean	SEM	Mean	SEM	Mean	SEM	Mean	SEM	Mean	SEM
Sham	1	0.088	1	0.37	1	0.061301	1	0.041472	1	0.040961	1	0.186201
25 cGy	0.908375	0.079	3.168656	0.65	0.854235	0.086047	0.816719	0.072355	0.775037	0.111171	1.072337	0.150981
100 cGy	0.794567	0.038	3.812445	0.56	1.031276	0.072731	0.863885	0.07759	0.995203	0.041628	1.041353	0.071963

Frontal	F344-DATFC		LEW-DATFC		F344-pAKTFC		LEW-pAKTFC	
	Mean	SEM	Mean	SEM	Mean	SEM	Mean	SEM
Sham	1	0.277535	1	0.085012	1	0.152326	1	0.331914
25 cGy	1.234748	0.074389	1.952601	0.305528	0.129112	0.039753	0.294244	0.086149
100 cGy	1.11939	0.127897	2.412912	0.277344	1.291155	0.390526	0.662775	0.079778

Parietal	F344-pAktPC		LEW-pAktPC		F344-AktPC		LEW-AktPC		F344-TOHPC		LEW-TOHPC	
	Mean	SEM	Mean	SEM	Mean	SEM	Mean	SEM	Mean	SEM	Mean	SEM
Sham	1	0.107955	1	0.077492	1	0.027815	1	0.073202	1	0.166089	1	0.158831
25 cGy	1.322728	0.0893	0.813161	0.144239	0.901291	0.042731	0.711377	0.154049	0.699668	0.077386	0.526873	0.058689
100 cGy	1.658644	0.144542	0.610839	0.10311	1.193758	0.176972	0.605436	0.068972	0.512321	0.037896	0.660762	0.088053
